# Discovery of co-occurring driver pathways in cancer

**DOI:** 10.1186/1471-2105-15-271

**Published:** 2014-08-09

**Authors:** Junhua Zhang, Ling-Yun Wu, Xiang-Sun Zhang, Shihua Zhang

**Affiliations:** National Center for Mathematics and Interdisciplinary Sciences, Academy of Mathematics and Systems Science, Chinese Academy of Sciences, Beijing, 100190 China

## Abstract

**Background:**

It has been widely realized that pathways rather than individual genes govern the course of carcinogenesis. Therefore, discovering driver pathways is becoming an important step to understand the molecular mechanisms underlying cancer and design efficient treatments for cancer patients. Previous studies have focused mainly on observation of the alterations in cancer genomes at the individual gene or single pathway level. However, a great deal of evidence has indicated that multiple pathways often function cooperatively in carcinogenesis and other key biological processes.

**Results:**

In this study, an exact mathematical programming method was proposed to *de novo* identify **co**-occurring **m**utated **d**river **p**athways (CoMDP) in carcinogenesis without any prior information beyond mutation profiles. Two possible properties of mutations that occurred in cooperative pathways were exploited to achieve this: (1) each individual pathway has high coverage and high exclusivity; and (2) the mutations between the pair of pathways showed statistically significant co-occurrence. The efficiency of CoMDP was validated first by testing on simulated data and comparing it with a previous method. Then CoMDP was applied to several real biological data including glioblastoma, lung adenocarcinoma, and ovarian carcinoma datasets. The discovered co-occurring driver pathways were here found to be involved in several key biological processes, such as cell survival and protein synthesis. Moreover, CoMDP was modified to (1) identify an extra pathway co-occurring with a known pathway and (2) detect multiple significant co-occurring driver pathways for carcinogenesis.

**Conclusions:**

The present method can be used to identify gene sets with more biological relevance than the ones currently used for the discovery of single driver pathways.

**Electronic supplementary material:**

The online version of this article (doi:10.1186/1471-2105-15-271) contains supplementary material, which is available to authorized users.

## Background

The pathogenesis of cancer in humans is still poorly understood. To improve the diagnosis and treatment of cancer patients, several large-scale cancer genomics projects (e.g., the Cancer Genome Atlas (TCGA) [1], and International Cancer Genome Consortium (ICGC) [2]) have been performed in recent years. Analyzing these high-throughput data provides valuable opportunities to understand the formation and progression of cancer [3, 4].

Generally, a large number of mutations occur in cancer genomes (e.g., somatic mutations and copy number alterations (CNAs)). One crucial step in cancer research is to distinguish driver mutations and driver genes, which contribute to the progression of cancer from normal to malignant states, from passenger mutations and passenger genes, which accumulate in cells but do not contribute to cancer development [5, 6]. Most early efforts were devoted to the detection of individual driver genes based on recurrent mutations of the genes in a large cohort of cancer patients [7].

Because of the mutational heterogeneity of cancer genomes, more attention has been paid to identify driver pathways and modules rather than individual genes in recent years [1, 8, 9]. It is noteworthy that most such methods involve the use of prior knowledge about pathways and/or protein interaction networks. For example, known pathways were analyzed for enrichment of somatic mutations [1, 8, 9], or were examined to find which ones are significantly disturbed across many patients [10, 11]. On the other hand, several studies indicated that driver pathways often cover a large number of samples. More importantly, mutations of the genes in one pathway usually exhibit mutual exclusivity, i.e., a single mutation is usually enough to disturb one pathway [12, 13]. These rules have been frequently used to detect driver pathways and modules [14–16]. For example, Ciriello *et al.* proposed MEMo (Mutual Exclusivity Modules) to detect oncogenic network modules within a constructed network using gene mutation information and a human reference network (including protein interactions and signal transduction pathways) [14].

However, it is believed that the human protein interaction network is incomplete. There are many unknown protein interactions and a great deal of knowledge about biological pathways remains unclear. Many reverse engineering approaches were developed in recent years to infer complex biological regulatory relationships. For example, Acharya *et al.* proposed the Gene Set Gibbs Sampling (GSGS) method to reconstruct signaling pathway structures by sequentially inferring the information flows from the overlapping information flow gene sets [17]. It is necessary to develop new methods to discover mutated driver pathways or core modules without relying on prior information. Recently, Vandin *et al.* proposed an approach, called Dendrix (*de novo* driver exclusivity), to *de novo* discover mutated driver pathways using somatic mutation data [18]. In this method, a novel weight function was introduced by combining the coverage and exclusivity of the gene set. Maximization of the weight function is defined as the maximum weight submatrix problem. This was originally solved by the Markov chain Monte Carlo (MCMC) method [18], and was then addressed using an exact binary linear programming (BLP) model [19]. However, these studies for the identification of driver pathways or core modules have all focused on single pathways or modules [15, 16, 18, 19]. How various cellular and physiological processes are coordinately altered during the initiation and progression of cancer, it is still a major challenge.

It is well known that multiple pathways with mutations are generally required for cancer [20]. In fact, it has been recently recognized that pathways often function cooperatively in carcinogenesis [13, 21–23]. Based on mutation data from COSMIC [24] and six major cancer-associated pathways from previous studies, Yeang *et al.* demonstrated that there were significant combinatorial patterns of mutations occurring in the same patients (i.e., co-occurring), for which the corresponding genes usually function in different pathways, whereas mutations in genes functioning in the same pathway are rarely mutated in the same sample (i.e., mutually exclusive) [13]. Cui *et al.* identified 12 oncogene-signaling blocks from the integrated human signaling network [21]. They found that some of them (such as the *RAS* and *TP53* blocks in central nervous system, pancreas, skin, and blood tumors) would collaboratively promote cancer signaling and foster tumorigenesis. Using 18 pathways enriched with mutations in lung adenocarcinoma [8], Gu *et al.* investigated pathway cooperation in cancer cells in terms of superpathways, which are clusters of co-disrupted pathways whose significance is tested by the hypergeometric model [25]. More recently, Gu *et al.* devised a heuristic approach to detect cooperative functional modules in the glioblastoma multiforme (GBM) altered network which is obtained by mapping mutated genes onto a protein interaction network from the Pathway Commons database, and several pairs of significantly co-altered modules were identified which are involved in the main pathways known to be perturbed in GBM [26].

All these studies indicate that carcinogenesis is a complex process and the malignant transformation from a normal cell to a tumor is indeed a highly cooperative procedure involving synergy between pathways. Therefore, systematically exploring the complex collaboration among different biological pathways and functional modules is a crucial step, which will shed new lights on our understanding of the cellular mechanisms underlying tumorigenesis. Current studies have mainly focused on the utilization of prior knowledge to determine whether two or more pathways or modules are simultaneously perturbed in the same samples. Considering the incompleteness of the knowledge about pathways and protein interaction networks, *de novo* discovery of collaborative pathways playing driver roles in cancer initiation and development is of pressing need. Although iteratively performing Dendrix [18] or BLP [19] can obtain multiple pathways by removing the gene sets found in each previous iteration, however, such pathways are not guaranteed to be significantly co-disrupted in the same patients.

In this study, a mathematical programming approach to discover **co**-occurring **m**utated **d**river **p**athways (CoMDP) in cancer generation and progression was developed. The co-occurring pathways detected here possess two properties: first, each pathway is a set of mutated genes with high coverage and high exclusivity; second, the mutations between pathway genes exhibit a statistically significant co-occurrence in cancer samples. CoMDP is an exact method where the optimal set of pathways is obtained using an efficient algorithm. It does not require any prior information besides mutation profiles. To evaluate this method, we first applied it onto simulated data and compared it with the original BLP method. Then we applied it onto four biological datasets and several pathways which might play collaborative roles in carcinogenesis were identified. For example, for the glioblastoma tumor data and lung adenocarcinoma data, several significant co-occurring gene pathways were detected. Each pair interacts and regulates the cell survival and protein synthesis processes. In addition, a modified form (named mod_CoMDP) was proposed in situations in which a certain pathway has been previously proven to play important roles in some cancers and one wants to know whether there are other pathways with cooperative effects in tumorigenesis. Furthermore, multiple co-occurring driver pathways can be discovered by combining previously detected pairs of gene sets and identifying others using mod_CoMDP. When applied to the ovarian carcinoma dataset, CoMDP and/or mod_CoMDP identified driver pathways related to *TP53* in the generation and progression of ovarian cancer. In summary, we developed a method for identifying mutated co-occurring driver pathways which can enhance the understanding of molecular mechanisms underlying tumorigenesis.

## Methods

### Brief introduction of the maximum weight submatrix problem

The Dendrix method was designed to *de novo* discover a single mutated driver pathway from somatic mutation data, where a weight function *W* was introduced by combining the coverage and exclusivity of the gene set [18]. Given a binary mutation matrix *A* with *m* rows (samples) and *n* columns (genes), the maximization of *W* was defined as the maximum weight submatrix problem [18], which means to find a submatrix *M* of size *m*×*k* in the mutation matrix *A* by maximizing the weight function *W*:
1

where *Γ*(*g*)={*i*:*A*_*ig*_=1} denotes the set of patients in which the gene *g* is mutated and *Γ*(*M*)=∪_*g*∈*M*_*Γ*(*g*), |*Γ*(*M*)| measures the coverage of *M* and  measures the coverage overlap of *M*.

**Figure 1:**
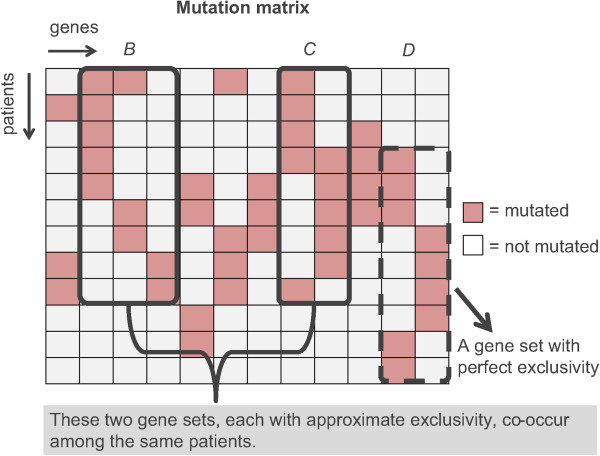
**Schematic illustration of driver gene sets (pathways) and their co-occurrence in the mutation matrix.** Three gene sets, *B*, *C*, and *D*, with high coverage and high exclusivity are shown. Gene sets *B* and *C* occur simultaneously among the same patients.

In addition to the stochastic MCMC search procedure [18], a BLP model has been introduced to solve this problem exactly [19]. Inspired by the BLP model, a binary linear programming model CoMDP to discover co-occurring driver pathways was developed here (Figure 1). The focus was placed on finding possible cooperative driver pathways in carcinogenesis. For example, in Figure 1, the gene set *D* not *C* can be detected using MCMC or BLP for *k*=2. This is because gene set *D* has higher mutation score *W* than that of gene set *C*. Mutations in the gene set *B* and the gene set *C* occurred simultaneously among a cohort of patients. CoMDP can successfully identify such co-occurring gene sets which may have been missed by previous approaches.

### CoMDP: a binary linear programming model for the identification of co-occurring driver pathways

For the mutation matrix *A*, let us consider two submatrices *M* and *N* (which correspond to two gene sets or pathways *S* and *T*). Given the coverage *Γ*(*M*) and *Γ*(*N*) of the two gene sets (sometimes called individual coverage in this study), we define (1) the common coverage ; (2) the union coverage . We further define the non-shared coverage *d*(*M*,*N*)=*b*(*M*,*N*)−*c*(*M*,*N*), which describes the extent of the mutation co-occurrence between the two gene sets: the smaller the value *d*, the larger the co-occurrence is. As suggested before, *ω*(*M*) and *ω*(*N*) reflect the exclusivity of *M* and *N* respectively.

To identify co-occurring gene sets with large coverage and high exclusivity, we introduce the following weight function *H*:
2

To maximize this weight function, a binary linear programming model is introduced as follows:
34

where *u*_*j*_ and *v*_*j*_ are indicators whether column *j* of *A* falls into the submatrx *M* or *N*, so all the columns *j*’s with *u*_*j*_=1 and *v*_*j*_=1 constitute *M* and *N* respectively; *x*_*i*_ and *y*_*i*_ are indicators whether the entries of row *i* of *M* and *N* are not all zeros, so  and  represent the coverage of *M* and *N* (i.e., |*Γ*(*M*)| and |*Γ*(*N*)|) respectively; *z*_*i*_ is the indicator whether both *x*_*i*_ and *y*_*i*_ equal to 1, so  represents the overlap between the coverage of *M* and *N* (i.e., the common coverage *c*(*M*,*N*)). *k* is the total number of genes within *S* and *T*; and finally, *λ* and *η* are two parameters controlling the common coverage *c*(*M*,*N*) and the non-shared coverage *d*(*M*,*N*) of the two gene sets.

Note that  and  in model (3) are always nonnegative according to the constraints in (4). One can properly set *λ* and *η* to be positive or negative to obtain gene sets with specific characteristics. For example, if *λ*<0 and *η*>0, the model tends to detect gene sets with large non-shared coverage but small common coverage under the maximization of *G*(*x*,*y*,*z*,*u*,*v*). Certainly, *λ*>0 and *η*<0 must be set if one wants to identify co-occurring driver pathways by maximizing the function *H* in (2), which is the main focus of this study. More discussion on the behavior of the model with *λ* and *η* can be referred to **Simulation study** below.

### mod_CoMDP: Finding a pathway that co-occurs with a known one

In some cases, some prior information is known for a disease. For example, a certain pathway may have been previously proven to play important roles in cancer. The problem is determining whether another pathway with a cooperative effect on tumorigenesis exists. CoDMP can be modified to answer this question to some extent. For a known pathway or a gene set *C*, a possible co-occurring pathway *D* can be identified by the following modified optimization problem:
56

where *x*_*i*_ and *u*_*j*_ are indicators whether the entries of row *i* in gene set *C* are not all zeros and whether the gene corresponding to column *j* of *A* falls into *C*, respectively; *y*_*i*_,*z*_*i*_,*v*_*j*_ and the parameters *λ* and *η* have the same meaning as in (3) and (4); *r* is the size of the desired gene set *D*.

Generally, a branch-and-bound algorithm or others can be used to produce an optimal exact solution for CoMDP (also for mod_CoMDP). In this study, an IBM ILOG CPLEX Optimizer was used to test the effectiveness of the model. The experiments were performed on a 2.50 GHz Core i5-2520M CPU PC. For each given *k*, CoMDP can automatically identify two gene sets when the sum of their sizes equals *k*. Although the problem was NP-hard, it can still be solved efficiently due to the sparsity of the mutation matrix.

### Statistical significance

A permutation test was used to assess the significance of the results. As in a previous study [18], the weight *W* in (1) served as a statistic to test the significance of the exclusivity and coverage of each identified gene set (called individual significance). We employed the co-occurrence ratio, which is defined as the ratio of the common coverage to the union coverage, as the statistic to test the significance of the co-occurrence of these two gene sets (called co-occurrence significance).

### Simulation data

Three datasets were constructed to illustrate the properties of the proposed method. The first set of simulated data, Sim _data1, was generated as in a previous study [19]. First, an empty *m* (samples) ×*n* (genes) matrix was given (*m*=500,*n*=1,000 were used). Then, gene sets *M*_*i*_ (*i*=1,⋯,*I*; and each set has 10 genes) with a mutation probability *p*_*i*_ were embedded in the matrix (*p*_*i*_=1−*i*·*Δ*, *Δ*=0.05, and *I*=10 were used here). For each sample, a gene uniformly chosen from *M*_*i*_ with *p*_*i*_ was mutated, and once one gene was mutated, the other genes in *M*_*i*_ had a probability *p*_0_ to be mutated (here *p*_0_=0.04 was used). Finally, the genes not in *M*_*i*_ were mutated in at most three samples. The second dataset, Sim _data2, was generated using the strategy described above for noisy probability *p*_0_ from 0.04 to 0.24 in steps of 0.02.

The third dataset, Sim _data3, was generated as follows. Starting with an empty *r*×*s* matrix (here *r*=600,*s*=1,000 were used), we embedded *J* gene sets *N*_1_,*N*_2_,⋯,*N*_*J*_ (*J*≥1, here *J*=9 was used) into it. *N*_*i*_ has size *m*_*i*_×*n*_0_ (in this study *n*_0_=5 and *m*_*i*_= [ *m*/2]+2^*i*−1^ were used where [*m*/2] denotes the integer part of *m*/2). *N*_*i*_ was constructed according to the strategy like that for *M*_*i*_ stated above. Similarly, we mutated the genes not in *N*_*i*_ at most in three samples.

We note that the average mutation rate for genes in a dataset in current simulation study is comparable to those of real datasets. For example, for Sim _data2 with the noisy probabilities of 0.04 and 0.24, each gene has an average mutation rate 0.0142 and 0.0274 respectively. For the four biological datasets (GBM1, GBM2, lung cancer and ovarian cancer) introduced in the following subsection, each dataset has an average mutation rate of 0.0658, 0.0416, 0.0206, and 0.0134 for each gene, respectively.

### Biological data

To assess the proposed methods for practical applications, four biological datasets were collected due to their popularity and abundant prior knowledge. Note that the CoMDP can be easily applied to other cancer mutation datasets.

The glioblastoma multiforme data 1 (GBM1) and the lung adenocarcinoma dataset were obtained directly from a previous study [18]. These sets contain mutations in 178 genes across 84 GBM patients (samples) and 356 genes in 163 lung cancer patients, respectively. The GBM2 and ovarian carcinoma datasets were obtained from another previous study [16]. These sets contain CNAs for 1269 genes spanning 169 GBM patients, somatic mutations for 343 genes across 135 GBM patients, CNAs in 966 genes across 559 ovarian patients, and somatic mutations in 8431 genes across 320 ovarian cancer patients. For the last two datasets, somatic mutations and CNAs were first integrated by merging the genes on the common patients. Finally, a binary mutation matrix *A* was obtained for each of the four datasets. The genes that are mutated in the same samples were combined into a gene set which was named as a metagene in this study. Note that the definition of a metagene differs from that defined based on the matrix factorization method.

## Results and discussion

### Simulation study

#### CoMDP can get the optimal solution of the original maximum weight submatrix problem

Like the BLP model [19], CoMDP can detect all the embedded gene sets in Sim _data1 with *k*=10, *η*=1 and *λ*<0 (here *λ*=−10 was used). In the current situation, CoMDP usually degenerates to find one gene set which corresponds to the optimal solution of BLP. Sometimes it can produce two gene sets with no common coverage. For example, with Sim _data1 where ten gene sets were embedded in the 500 (samples) × 1000 (genes) matrix, we identified two sets with two and eight genes respectively, which are mutated in 74 and 340 samples respectively. But their common coverage is 0. In fact, these two sets constitute one of the embedded gene sets which can be found using the BLP method, so they can be viewed as the same driver gene set as obtained using BLP directly.

**Figure 2:**
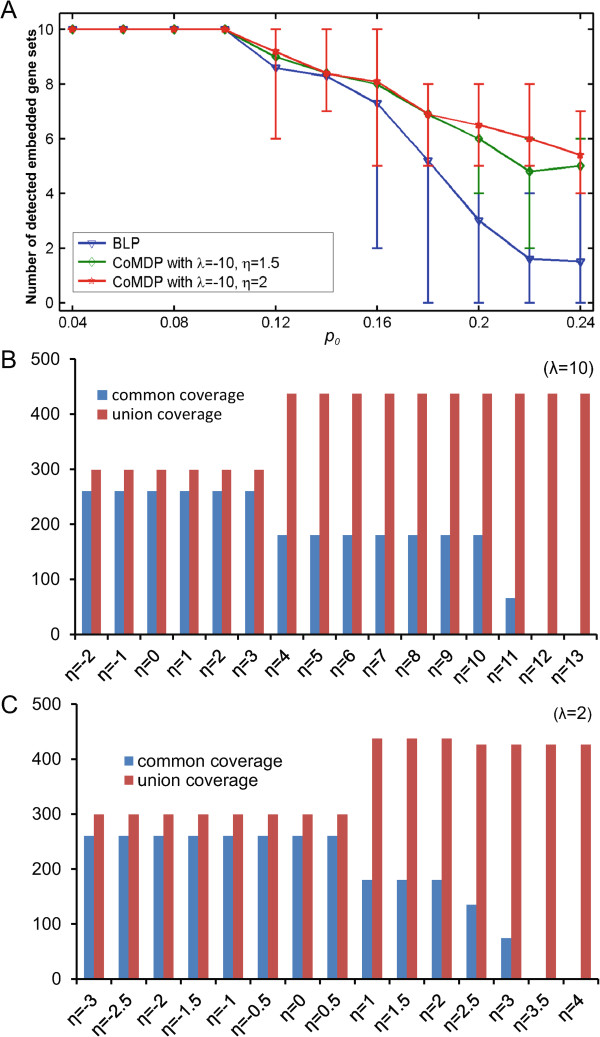
**Results of CoMDP applied to simulation data.** **(A)** Comparison of CoMDP with BLP for the ability to identify the embedded gene sets. Display of co-occurrence of the gene sets detected by CoMDP for different *η* when *λ*>0: **(B)** *λ*=10, and **(C)** *λ*=2.

Note that as *p*_0_ increases, the exclusivity among the genes in *M*_*i*_ decreases, so the detection of the embedded gene sets *M*_*i*_ becomes more and more difficult. Let *k*=10. CoMDP (*λ*=−10, *η*=1) and BLP were applied on Sim _data2. Both were able to precisely identify all ten embedded gene sets when *p*_0_≤0.10. For BLP, the average number of detected embedded gene sets decreased sharply as *p*_0_ increased. However, by properly choosing the parameter *η*, CoMDP can obtain more accurate and robust results than BLP at high values of *p*_0_. For example, CoMDP with *λ*=−10 and *η*=2 has much higher identification accuracy than BLP for *p*_0_≥0.20 (Figure 2A).

## CoMDP can identify co-occurring gene sets efficiently

We further applied CoMDP to Sim _data3 to demonstrate its effectiveness, and assessed the effect of *λ* and *η*. We found that the results are robust with the selection of these two parameters. CoMDP can always get the embedded gene sets with the largest co-occurrence ratio 0.8696 for *λ* ranging from 6 to 24 in step of 2 and *η* ranging from -10 to -1 in step of 1. The performance of CoMDP was also demonstrated on different *η* with *λ*=10 (Figure 2B). For example, when *η*≤3, the two detected 5-gene sets mutated almost in the same samples (the individual coverage of these two sets was 286 and 273 and common coverage was 260). When *η* became larger the coverage difference of the two sets increased, and the common coverage became smaller. When *η*≥12, CoMDP detected one gene set with 10 genes, which had the coverage 437 and so the co-occurrence ratio was 0. Generally speaking, we found that CoMDP has similar performance when *λ* equals 2 or 10 (Figure 2C). A high co-occurrence ratio (i.e., 0.8696) was obtained when *η*≤0.5, and a ratio of 0 was obtained when *η*≥3.5. The simulation study confirmed that *λ*>0 and *η*<0 are the proper selections for identifying co-occurring gene sets. In the following biological applications, without loss of generality, *λ*=10 and *η*=−2 were used.

## Applications to biological data

In this section, CoMDP was used on four biological datasets (i.e., GBM1, lung cancer, GBM2, and ovarian carcinoma datasets) to identify the co-occurring driver pathways with *k*=4∼10. We also demonstrated that mod_CoMDP (model (5) and (6)) was applied onto the ovarian carcinoma data to detect more driver pathways co-occurred with *TP53* in carcinogenesis and to find multiple significant co-occurring driver pathways. Each run for GBM1 and GBM2 datasets takes less than two seconds, and each run for the lung cancer dataset takes less than four seconds.

### GBM1 dataset

**Figure 3:**
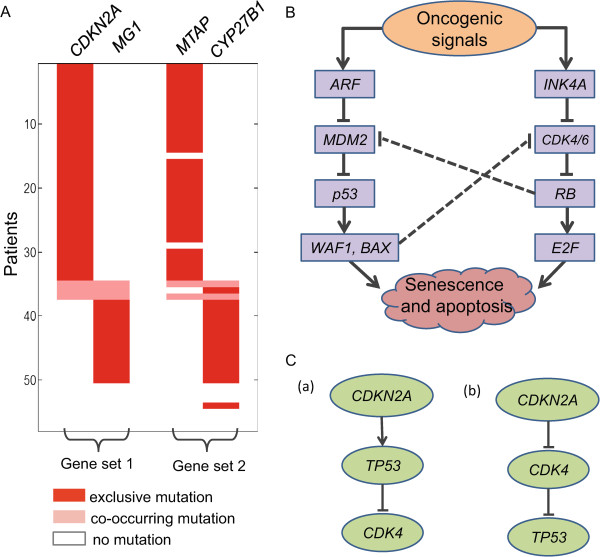
**The results of CoMDP on the GBM1 dataset with** ***k*** **=4 and** ***k*** **=5, and the related** ***p53*** **and** ***RB*** **pathways.** **(A)** The co-occurrence between the two gene sets {*CDKN2A*, *M* *G*_1_} and {*MTAP, CYP27B1*} identified by CoMDP with *k*=4 in the GBM1 dataset. **(B)** The *TP53* and *RB* tumor suppressor pathways. *ARF* and *INK4A* are two alternatively spliced transcripts of *CDKN2A*. This figure was adapted/extracted according to [31, 32]. **(C)** The alterative gene regulatory pathway ((a) or (b)) involving *CDKN2A, CDK4* and *TP53*.

For *k*=4, two gene sets were detected: {*CDKN2A, MG*_1_} and {*MTAP, CYP27B1*} (*MG*_1_ is a metagene consisting of *CDK4, FAM119B, MARCH9, TSFM, CENTG1, METTL1* and *TSPAN31*) with individual significance *p*_1_=0.0207, *p*_2_=0.0058, co-occurrence significance *p*_1,2_<0.0001, and co-occurrence ratio *r*_1,2_=0.9412 (Table 1). The two genes *MTAP* and *CDKN2A* were found to be frequently co-deleted [27, 28]. They are both located on chromosome 9p21, a typical tumor suppressor region whose deletion is related to many different types of cancers. *CYP27B1* and the metagene *MG*_1_ were mutated in the same patients with one exception: a single-nucleotide mutation was recorded in one additional patient for *CYP27B1* (Figure 3A). Previous studies have suggested that *CDK4* is the target of a common CNA in the corresponding patients [29]. Two protein products of *CDKN2A*, *INK4A* (also known as *p16*) and *ARF* (also known as *p14*), are involved in the *p53* and *RB* tumor suppressor pathways (Figure 3B). It has been shown that any error disrupting these pathways causes tumor formation [30]. *CDKN2A* and *CDK4* are considered part of the *RB* pathway. Both *MTAP* and *CYP27B1* encode important enzymes. The enzymes encoded by *MTAP* play a major role in polyamine metabolism and those encoded by *CYP27B1* play a role in calcium metabolism and tissue differentiation.

**Table 1:** **Co-occurring gene sets identified by applying CoMDP to GBM1**

k	Gene set 1	Gene set 2	***p***_1_	***p***_2_	***n***_1_	***n***_2_	***r***_1,2_	***p***_1,2_
4	*CDKN2A*, *M* *G*_1_	*MTAP, CYP27B1*	0.0207	0.0058	50	49	0.9412	<0.0001
	*CDKN2A, TP53,*							
5	*M* *G*_1_	*CDKN2B, CYP27B1*	0.0003	0.0018	68	57	0.7606	<0.0001
	*CDKN2A, PTEN,*	*CDKN2B, TP53,*						
6	*CYP27B1*	*M* *G*_1_	0.0002	0.0003	69	71	0.8182	<0.0001
	*CDKN2A, PTEN,*	*CDKN2B, RB1,*						
7	*CYP27B1*	*TP53*, *M* *G*_1_	0.0002	0.0001	69	74	0.8571	<0.0001
	*CDKN2A, PTEN,*	*CDKN2B, RB1,*						
8	*NF1, CYP27B1*	*M* *G*_1_, *ERBB2*	0.0015	<0.0001	72	70	0.8933	<0.0001
	*CDKN2A, PTEN, NF1,*	*CDKN2B, RB1,*						
9	*CYP27B1, KDR*	*M* *G*_1_, *ERBB2*	0.0006	<0.0001	74	70	0.9200	<0.0001
	*CDKN2A, PTEN, CYP27B1,*	*CDKN2B, NF1, RB1,*						
10	*KDR*, *M* *G*_2_	*M* *G*_1_, *ERBB2*	<0.0001	<0.0001	72	73	0.9333	<0.0001

Here *p*_1_ and *p*_2_ are the *p*-values of the individual significance of two identified gene sets, *p*_1,2_ represents the *p*-value of their co-occurrence significance, *n*_1_ and *n*_2_ denote their respective coverage, and *r*_1,2_ is the ratio of the common coverage to their union coverage (i.e., co-occurrence ratio). There are same meanings in the following tables. *MG*_1_ is a metagene including seven genes: *CDK4, FAM119B, MARCH9, TSFM, CENTG1, METTL1, TSPAN31*. *MG*_2_ is a metagene including four genes: *WT1, SLC1A2, PAX6, ABCC4*.

For *k*=5, two gene sets including {*CDKN2A, TP53, MG*_1_} and {*CDKN2B, CYP27B1*} were detected with *p*_1_=0.0003, *p*_2_=0.0018, *p*_1,2_<0.0001 and *r*_1,2_=0.7606. *CDKN2B* encoding *INK4B* (also known as *p15*) also locates in chromosome 9p21 homozygous deletion region, and *CDKN2B* is usually co-deleted with *CDKN2A*. This disrupts the *p53* and *RB* pathways. For this reason, combinatorial inactivation of *CDKN2A* and *CDKN2B* is frequently observed in these tumors. The cross-talk between the *p53* and *RB* pathways (Figure 3B) suggests that *CDKN2A, TP53* and *CDK4* are in the same pathway (Figure 3C(a) or 3C(b)).

**Figure 4:**
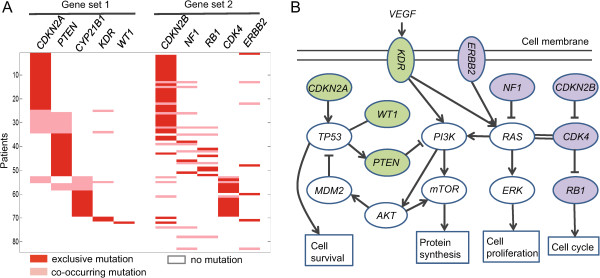
**The results of CoMDP on the GBM1 dataset with** ***k*** **=10.** **(A)** The co-occurrence between the two gene sets {*CDKN2A, PTEN, CYP27B1, KDR, WT1*} and {*CDKN2B, NF1, RB1, CDK4, ERBB2*}. **(B)** Several pathways are involved, including the *p53*, *PI3K/Akt*, *RTK/RAS/ERK*, *RB* and *RTK/RAS/PI3K* signaling pathways. The line between *p53* and *WT1* denotes they are physically interacted, whereas the double lines between *KRAS* and *CDK4* represents sometimes there is a synthetic lethal interaction between them. The green and blue nodes denote the genes involved in the two detected co-occurring gene sets. The regulatory relations are extracted from the KEGG database and related literature.

For *k*=10, two gene sets {*CDKN2A, PTEN, CYP27B1, KDR, MG*_2_} and {*CDKN2B, NF1, RB1, MG*_1_, ERBB2} with *p*_1_,*p*_2_ and *p*_1,2_ less than 0.0001 and *r*_1,2_=0.9333 were identified (Table 1 and Figure 4A). The first gene set was found to be involved in the *p53* and *PI3K/Akt* signaling pathways and the second in the *RB* and *RTK/RAS/ERK* signaling pathways. The *RTK/RAS/PI3K* signaling pathway can also be induced by the mutations in these two gene sets (Figure 4B). These pathways are implicated in biological processes associated with cell survival, cell cycle, protein synthesis, and cell proliferation. *p53*, *RB*, and *RTK/RAS/PI3K* have been previously reported to contribute to GBM pathogenesis in original TCGA GBM studies [1]. Five well-known tumor suppressors (*CDKN2A, CDKN2B, PTEN, NF1, and RB1*) are involved in these two gene sets. Besides the co-occurrence of *CDKN2A* and *CDKN2B*, *NF1* and *RB1* in the second gene set have exclusive mutations, which are co-occurrent with mutations of *PTEN* in the first set (Figure 4A). Recently, several studies have shown the cooperativity of tumor suppressors in carcinogenesis [33–35]. For example, Rahrmann *et al.* demonstrated that co-occurring mutations in *PTEN* and *NF1* cooperate in the development of grade 3 PNSTs (peripheral nerve sheath tumors) in mice, suggesting that they may cooperate in human MPNST (malignant PNST) progression [33]. Another study by Chow *et al.* [34] showed that cooperativity among *PTEN*, *TP53*, and *RB1* can cause high-grade astrocytoma in mouse adult brain, in which the majority of glioblastomas arise. For another two almost simultaneously mutated oncogenes *CYP27B1* and *CDK4* (Figure 4A), Beckner *et al.* have demonstrated their cooperative amplification and co-expression for potential modulation of vitamin D in glioblastomas [36].

On the other hand, *WT1* encodes a transcription factor that plays an essential role in cellular development and cell survival [37]. It regulates the expression of numerous target genes, including the famous tumor suppressor *TP53* and the *Wnt* signaling pathway [38]. *KDR* encodes a *VEGF* (vascular endothelial growth factor) receptor. *VEGF* plays a crucial role in angiogenesis and progression of malignant brain tumors. *ERBB2* encoding the protein *HER2* (human epidermal growth factor receptor 2) is a member of the epidermal growth factor receptor (*EGFR*) family, and it has been shown to play an important role in the pathogenesis and progression of many different types of cancer. *WT1, KDR* and *ERBB2* may drive the carcinogenesis of GBM, indicating that CoMDP can identify low-frequency candidate driver genes that play important roles in cancer initiation and development.

### Lung cancer

In this case, the significant results were obtained with *k*=4,5,10 (Table 2). For *k*=4 the co-occurring gene sets are {*ATM, TP53*} and {*EGFR, KRAS*}, and for *k*=5 they are {*ATM, TP53*} and {*EGFR, KRAS, STK11*}. As stated in a previous study [18], *ATM* and *TP53* interact directly and are involved in the cell cycle checkpoint control [39]. *EGFR, KRAS* and *STK11* are all involved in the regulation of the *mTOR* signaling pathway, whose dysregulation has been reported to be important to lung adenocarcinoma [8]. However, the gene set {*ATM, TP53*} can only be obtained by removing the mutations of {*EGFR, KRAS, STK11*} from the dataset in the previous study by Vandin [18]. Here, these two gene sets were identified simultaneously and found to show significant co-occurrence. *ATM, TP53* are involved in the regulation of cell apoptosis and *EGFR, KRAS, STK11* are related to protein synthesis, indicating that the cooperativity of these two processes for the generation and progression of lung cancer.

**Table 2:** **Co-occurring gene sets identified by applying CoMDP to the lung cancer data**

k	Gene set 1	Gene set 2	***p***_1_	***p***_2_	***n***_1_	***n***_2_	***r***_1,2_	***p***_1,2_
4	*ATM, TP53*	*KRAS, EGFR*	0.0121	<0.0001	76	90	0.3833	0.0129
5	*ATM, TP53*	*STK11, KRAS, EGFR*	0.0125	<0.0001	76	110	0.4091	0.0220
	*STK11, ATM,*	*KRAS, NTRK3, EGFR,*						
10	*TP53, PAK4*	*GNAS, EPHA3, NRAS*	0.0379	<0.0001	98	108	0.5489	0.0001

For *k*=10, {*STK11, ATM, TP53, PAK4*} and {*KRAS, NTRK3, EGFR, GNAS, EPHA3, NRAS*} were identified. All four genes *STK11, ATM, TP53, PAK4* have been demonstrated to be closely related to the *p53* signaling pathway in lung cancer [40, 41]. Two members of the *RAS* subfamily, *KRAS* and *NRAS* function as binary molecular switches controlling the intracellular signaling networks that regulate several key cancer-related processes, such as proliferation, differentiation, cell adhesion, apoptosis, and cell migration. *GNAS* is a guanine nucleotide-binding protein (G protein). It acts as a modulator or transducer in various transmembrane signaling systems. *GNAS* may interact with *MDM2*, which may lead to *MDM2*-mediated degradation of *TP53*. Solomon *et al.* found that many kinds of *TP53* mutations can regulate *RAS* in different ways, inducing a cancer-related gene signature [42]. Kosaka *et al.* demonstrated that *TP53, EGFR* and *KRAS* may cooperatively determine the prognosis of the patients in lung adenocarcinoma [43].

### GBM2 dataset

**Figure 5:**
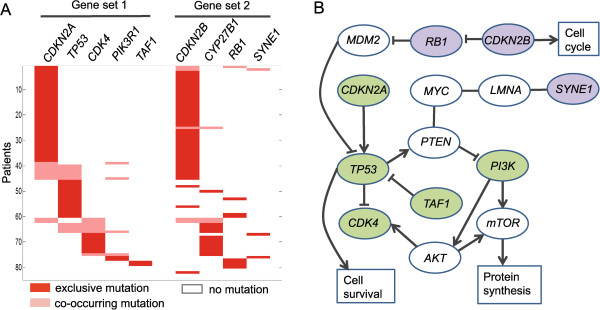
**The results of CoMDP on the GBM2 dataset with** ***k*** **=9.** **(A)** The significant co-occurrence mutations between the two gene sets {*CDKN2A, TP53, MG*_3_ *, PIK3R1, TAF1*} and {*CDKN2B, CYP27B1, RB1, SYNE1*}. **(B)** The relevant pathways of the two gene sets.

We observed that some new co-occurring gene sets were identified for GBM2 compared to GBM1 (Table 3). For *k*=9, we identified {*CDKN2A, TP53, MG*_3_*, PIK3R1, TAF1*} and {*CDKN2B, CYP27B1, RB1, SYNE1*} (*MG*_3_ is a metagene including *CDK4, MARCH9* and *TSPAN31*) with *p*_1_=0.0002, *p*_2_<0.0001, *p*_1,2_<0.0001 and *r*_1,2_=0.8642 (Figure 5A and Table 3). In addition to the cooperative effects between *CDKN2A* and *CDKN2B*, *TP53* and *RB1* have been reported to have frequently co-occurring mutations related to several cancers, including the central nervous system tumor [13]. Recently, the collaboration of *TP53* and *CDKN2B* was also studied with respect to cell apoptosis and aneurysm formation [44]. On the other hand, for the two detected low-frequency mutated genes *TAF1* (2/170) and *SYNE1* (3/170), *TAF1* encoding a transcription initiation factor phosphorylates *TP53* during G1 cell-cycle progression, so *TAF1* may be a member of the *p53* signaling pathway; *SYNE1* was found to be associated with the GBM patients’ lifetime, and was therefore considered to be an important biomarker of glioblastoma survival [45]. Our studies indicated that the *p53*, *RB*, and the *PI3K*-related signaling pathways may collaboratively contribute to carcinogenesis in GBM via combined genetic alterations (Figure 4B and Figure 5B).

**Table 3:** **Co-occurring gene sets identified by applying CoMDP to GBM2 with somatic mutations and CNAs**

k	Gene set 1	Gene set 2	***p***_1_	***p***_2_	***n***_1_	***n***_2_	***r***_1,2_	***p***_1,2_
4	*CDKN2A,* *M* *G*_3_	*CDKN2B, CYP27B1*	0.0045	0.0056	60	63	0.9219	<0.0001
	*CDKN2A,*							
5	*CYP27B1, COL1A2*	*CDKN2B,* *M* *G*_3_	0.0072	0.0018	62	63	0.9531	<0.0001
	*CDKN2A,*	*CDKN2B,*						
6	*CYP27B1, COL1A2*	*M* *G*_3_, *ERBB2*	0.0073	0.0003	62	65	0.9538	<0.0001
	*CDKN2A, TP53,*	*CDKN2B, RB1,*						
7	*M* *G*_3_ *, TAF1*	*CYP27B1*	0.0001	0.0001	77	70	0.8375	<0.0001
	*CDKN2A, TP53,*	*CDKN2B, RB1,*						
8	*M* *G*_3_ *, TAF1*	*CYP27B1, PRNP*	0.0002	<0.0001	77	71	0.8500	<0.0001
	*CDKN2A, TP53, TAF1,*	*CDKN2B, RB1,*						
9	*M* *G*_3_ *, PIK3R1*	*CYP27B1, SYNE1*	0.0002	<0.0001	79	72	0.8642	<0.0001
	*CDKN2A, TP53, TAF1,*	*CDKN2B, RB1, SYNE1,*						
10	*M* *G*_3_ *, PIK3R1*	*CYP27B1,* *M* *G*_4_	0.0001	<0.0001	79	73	0.8765	<0.0001

*MG*_3_ is a metagene including three genes: *CDK4, MARCH9, TSPAN31*. *MG*_4_ is a metagene including 168 genes.

### Ovarian cancer

The mutation distribution among genes in the ovarian carcinoma data is quite nonuniform. Among all the 314 samples, *TP53* is mutated in 251 of them and all the other genes are mutated in less than 26% of samples. This indicates that *TP53* plays a crucial role in the carcinogenesis of ovarian cancer (*TTN* was removed in the present analysis because of the possible artifacts of its mutations [46]). Determining whether there are other driver genes or pathways collaborating with *TP53* will be helpful for understanding the pathogenesis of this cancer.

We applied CoMDP to the ovarian cancer data with *k*=4∼10 (Table 4). The first three rows in Table 4 showed significantly co-occurring gene sets with *TP53*. For *k*=5 we identified {*MYC, CCNE1, NINJ2,MG*_5_} (*MG*_5_ is a metagene including *CHKB* and *KLHDC7B*). *MYC* and *CCNE1* are two important proto-oncogenes involved in cell cycle progression. The functional correlation of *MYC* and *TP53* in the carcinogenic progression of ovarian carcinoma and other cancers have been evaluated in several studies [47–49]. Recently, Kuhn *et al.* discovered that molecular genetic aberrations of *CCNE1* together with those of the *p53* and *PI3K* pathways are major mechanisms in the development of uterine serous carcinoma [50]. Both *CHKB* and *KLHDC7B* are located on chromosome 22q13.33, where *KLHDC7B* is involved in breast cancer and lymph node metastasis in cervical cancer and *CHKB* encodes choline kinase (*ChoK*) beta. de Molina *et al.* demonstrated that *ChoK* acts as a link connecting phospholipid metabolism and cell cycle regulation [51]. It is here supposed that *TP53* and *CHKB* may regulate CDK4/6 collaboratively to suppress the progression of ovarian cancer.

**Table 4:** **Co-occurring gene sets identified by applying CoMDP to the ovarian carcinoma dadaset**

k	Gene set 1	Gene set 2	***p***_1_	***p***_2_	***n***_1_	***n***_2_	***r***_1,2_	***p***_1,2_
4	*TP53*	*MYC, CCNE1, NINJ2*	1.0000	<0.0001	251	155	0.4397	0.0410
		*MYC, CCNE1,*						
5	*TP53*	*NINJ2,* *M* *G*_5_	1.0000	0.0100	251	169	0.4894	0.0250
		*MYC, CCNE1,*						
6	*TP53*	*NINJ2, ZNF596, USH2A*	1.0000	<0.0001	251	183	0.5228	0.0120
		*MYC, CCNE1,*						
7	*TP53, LYRM5*	*NINJ2, ZNF596, USH2A*	0.0270	0.0030	264	183	0.5629	<0.0001
		*MYC, CCNE1, NINJ2,*						
8	*TP53, LYRM5*	*BRD4, ZNF596, USH2A*	0.0230	0.0210	264	197	0.6007	0.001
		*MYC, CCNE1, NINJ2,*						
9	*TP53, LYRM5*	*BRD4, ZNF596,*	0.0340	0.0160	264	206	0.6263	<0.0001
		*USH2A, HMCN1*						
		*MYC, CCNE1, NINJ2,*						
10	*TP53, LYRM5*	*NF1, ZNF596,*	0.0390	0.001	264	211	0.6493	<0.0001
		*USH2A, HMCN1, TPD52L2*						

*MG*_5_ is a metagene including two genes: *CHKB, KLHDC7B*. For *k* = 4 ∼ 6 because of only one gene contained in the gene set 1, the corresponding *p*-value equals 1.0000.

To identify other driver gene sets coupled to *TP53*, we applied mod_CoMDP with *r*=1∼10 to the ovarian cancer data and significant results were obtained for *r*=3∼10 (Additional file 1: Table S1). For example, for *r*=10 we identified {*MYC, CCNE1, NINJ2,MG*_5_*, USH2A, NF1, HMCN1, ZNF596, USP35,MG*_6_} (*MG*_6_ is a metagene including four genes: *STMN3, SLC2A4RG, ZGPAT, RTEL1*) with *ra*=0.6563 (the co-occurrence ratio with *TP53*). Frequent somatic mutations in *NF1* have been previously shown to co-occur with *TP53* mutations in ovarian carcinomas [52, 53]. *STMN3* and *NF1* have been demonstrated to be involved in the *MAPK* signaling pathway [19]. Furthermore, to discover possible collaborations of multiple driver pathways with *TP*53, we combined *TP53* and the aforementioned 10-gene set into one nominal gene, which was considered mutated in a sample if both sets were mutated in that sample. Then we applied mod_CoMDP to identify gene sets significantly co-occurring with the nominal gene. For *r*=1∼10 we identified *PPP2R2A*, which is generally implicated in the negative control of cell growth and division. Kalev *et al.* revealed that *PPP2R2A* plays a critical role in DNA double-strand break repair through modulation of *ATM* phosphorylation [54]. Youn and Simon recently studied mutator alterations relevant to ovarian cancer [55]. Besides the well-known mutator gene *TP53*, they identified *PPP2R2A* and the chromosomal region 22q13.33 as the new mutator candidates. We find that these so called mutator genes, which increase genomic instability when altered, may be collaboratively involved in the processes of DNA synthesis and repair, chromosome segregation, damage surveillance, cell cycle checkpoints, and apoptosis. The discovered driver patterns here may provide new information to enhance our understanding of the ovarian carcinoma pathogenesis, and further explorative analysis is needed to verify their biological relevance.

## Conclusions

In this study, we proposed a method CoMDP for the *de novo* identification of co-occurring driver pathways in cancer. It considers two types of optimization simultaneously: First, it makes the maximization of the weight *W* for each individual pathway, i.e., high coverage and high exclusivity. Second, it ensures that the maximization of the inter-overlap between the pathway pair. Simulation study indicated that for a range of values of the parameters *λ* and *η*, CoMDP can always get the exact solution. It was here demonstrated that CoMDP has the following characteristics: (1) It can identify individual driver gene sets as BLP [19] or Dendrix [18]. (2) It obtains more accurate and robust results when the noise increases. (3) It uses no prior information such as the incomplete knowledge about the pathways and protein interaction networks. (4) CoMDP is an exact method and the procedure is quite fast.

When the project approximated to the end, we noticed that Leiserson *et al.* proposed a method for the simultaneous identification of multiple driver pathways [56]. The present study is related to Leiserson’s but in many ways quite different. First, the so-called Dendrix _*ILP*_ in [56] is the same as the BLP method [19]. Second, the weight function used by their Multi-Dendrix algorithm does not explicitly incorporate co-occurrence of mutations between genes in different pathways [56]. The Multi-Dentrix of the two gene sets was found to be a special form of the present model with *λ*=2 and *η*=1. Our simulation study demonstrated that, in this case, the coverage of the two sets detected was 286 and 331 respectively. Although the union coverage got larger (i.e., 437), a lower co-occurrence ratio 0.4119 was obtained because of the smaller common coverage. This also indicates that the multiple driver pathways (gene sets) identified by Multi-Dendrix cannot be guaranteed to be co-occurring.

We note that the heterogeneity among tumors can affect the findings of the current method. Investigating combinatorial patterns of driver pathways in different subtypes will be helpful for understanding the molecular mechanisms of carcinogenesis and designing efficient treatments for cancer patients. It will be interesting to explore the effect of heterogeneity among tumors in the future studies.

In summary, we have developed a method to identify co-occurring driver pathways, which may reveal the functional cooperation of different driver pathways during carcinogenesis. The results of this study show that the present method will be a powerful tool to explore the collaborative effects among mutated driver pathways and enhance our understanding of the molecular mechanisms.

## Electronic supplementary material

Additional file 1: **The results by applying mod_CoMDP to the ovarian carcinoma dadaset.** (PDF 24 KB)
