# Supplementary material for: Discovery of co-occurring driver pathways in cancer
Source: BMC Bioinformatics. 2014 Aug 9;15(1):271. doi: 10.1186/1471-2105-15-271 (PMC4133618; doi:10.1186/1471-2105-15-271)
Supplement: Supplementary file 1 — Additional file 1: The results by applying mod_CoMDP to the ovarian carcinoma dadaset. (PDF 24 KB) [file 12859_2014_6533_MOESM1_ESM.pdf]

Additional Files

Additional file 1 — The results by applying mod_CoMDP to the ovarian carcinoma dadaset

Table S1 - The gene sets co-occurrent with TP53 identified by applying mod_CoMDP to the ovarian carcinoma dadaset

r	Gene set	p	n	ra	pr
3	*MYC, CCNE1, NINJ2*	< 0.0001	155	0.4397	0.0430
4	*MYC, CCNE1, NINJ2,* MG_5	0.0060	169	0.4894	0.0280
5	*MYC, CCNE1, NINJ2, ZNF596, USH2A*	0.0020	183	0.5228	0.0190
6	*MYC, CCNE1, NINJ2, ZNF596, USH2A, BRD4*	0.0240	197	0.5610	0.0340
7	*MYC, CCNE1, NINJ2, ZNF596, USH2A, BRD4, HMCN1*	0.0220	206	0.5868	0.0060
8	*MYC, CCNE1, NINJ2, ZNF596, USH2A, NF1, HMCN1, TPD52L2*	0.0020	211	0.6098	0.0040
9	*MYC, CCNE1, NINJ2, ZNF596, USH2A, NF1, HMCN1, TPD52L2, TRIT1*	< 0.0001	221	0.6276	0.0010
10	*MYC, CCNE1, NINJ2,* MG_5*, USH2A, NF1, HMCN1, ZNF596, USP35,* MG_6	0.0310	226	0.6563	0.0010

Here p and pr denote the p-values of the significance of the detected gene set and its co-occurrence significance with *TP53* respectively, n denotes the gene set's overage, and ra denotes its co-occurrence ratio with *TP53*. MG_6 is a metagene including four genes: *STMN3, SLC2A4RG, ZGPAT, RTEL1.*
